# Healing of Extensive Through-and-Through Periradicular Lesion Using Apicoectomy Without Bone Grafting: A Case Report With 3-Year Recall

**DOI:** 10.1155/crid/7225338

**Published:** 2025-04-22

**Authors:** Qamar Hashem

**Affiliations:** Conservative Dentistry Department, College of Dentistry, Prince Sattam Bin Abdulaziz University, Al-Kharj, Saudi Arabia

**Keywords:** apical surgery, bone graft, EMS, endodontic surgery, high altitude, through-and-through lesions

## Abstract

The pathogenic process of through-and-through lesions leads to erosion and loss of both cortical plates. Endodontic microsurgery (MS)with bone graft and membrane placement (guided tissue regeneration (GTR)) is the proposed treatment for such cases. This article is aimed at discussing an unusual treatment protocol for a large through-and-through lesion without the use of bone grafting or membrane. A 26-year-old Air Force pilot traveling at high altitudes presented to the Endodontic division for management of pain and swelling related to the upper left anterior lesion concerning Teeth #21, #22, and #23. Following a thorough clinical examination, medical history, and clinical and radiographic examination, the diagnosis of the presence of a through-and-through lesion related to previously treated teeth. The treatment plan included endodontic MS without the use of GTR due to his work nature. Over 3 years, routine follow-ups were essential for tracking the damaged area's healing process, which ultimately resulted in full recovery. This case emphasizes how crucial it is to obtain a thorough history and use a multidisciplinary approach to diagnose and manage through-and-through lesions, achieving positive outcomes.

## 1. Introduction

Traditional root canal treatment (RCT) is aimed at eliminating microorganisms from the root canal system and creating effective barriers to prevent recontamination of the roots [1], thus healing most periapical radiolucencies [1]. Successful endodontic therapy requires thoroughly cleaning, shaping, and filling the entire root canal system. Triburcio et al. [2] reported that around 52% of adults have at least one tooth with periapical periodontitis, of which 3% are present in untreated teeth and 39% have persistent lesions in previously treated teeth. Persistence or secondary intra or extraradicular infections are the main cause of persistent lesions. Newly arising or recurrent periapical periodontitis, also known as posttreatment periapical periodontitis, results from the failure of primary treatment [3, 4]. Treatment options include root canal retreatment (ReRCT) [3], apicoectomy, or tooth extraction [5]. In cases where conventional treatment has not effectively resolved a periapical lesion, retreatment may be recommended as the initial therapeutic approach [4, 6, 7]. Endodontic surgery is recommended when nonsurgical retreatment is not possible or unlikely to improve a previous result [8].

Three categories of endodontic microsurgery (EMS) have been described: simple, complex, and periodontal. Complex cases include large lesions or through-and-through lesions (TTLs), which are solitary endodontic lesions without periodontal involvement. TTLs are either surgically created or due to the pathological resorption of both cortical plates [9, 10]. EMS is a surgical procedure to heal periradicular pathosis by accessing the root canal while preserving the periodontium, allowing a direct view of the periradicular surgical site [11–13]. The surgeon removes the diseased tissue, cleans out the canal system, and seals the defect [14]. Before the use of an operating microscope in endodontic surgery, the success rate was only 37.4% [15], lower than the success rate of nonsurgical retreatment [16].

However, the introduction of a modern technique that includes the use of a dental operating microscope, microinstruments, ultrasonic tips, and more biologically acceptable root-end filling materials [1, 17] has increased the success rate to approximately 79%–100% [18]. This shows a significant improvement in the success of microsurgical approaches.

This article discusses an unusual treatment protocol for a large TTL without using bone grafting or a membrane.

## 2. Case Report

A general dentist referred a 26-year-old male pilot to the endodontic division for the treatment of multiple teeth in the left anterior region. The patient, who was ASA1 with no medical history, was complaining of persistent pain in the upper left anterior lesion. Six months ago, a consultant endodontist performed endodontic retreatment for Teeth #22, #23, #24, and #25, followed by a permanent restoration. However, pain symptoms returned 2 weeks after treatment, which affected his daily activities and prompted him to visit the dentist.

**Figure 1:**
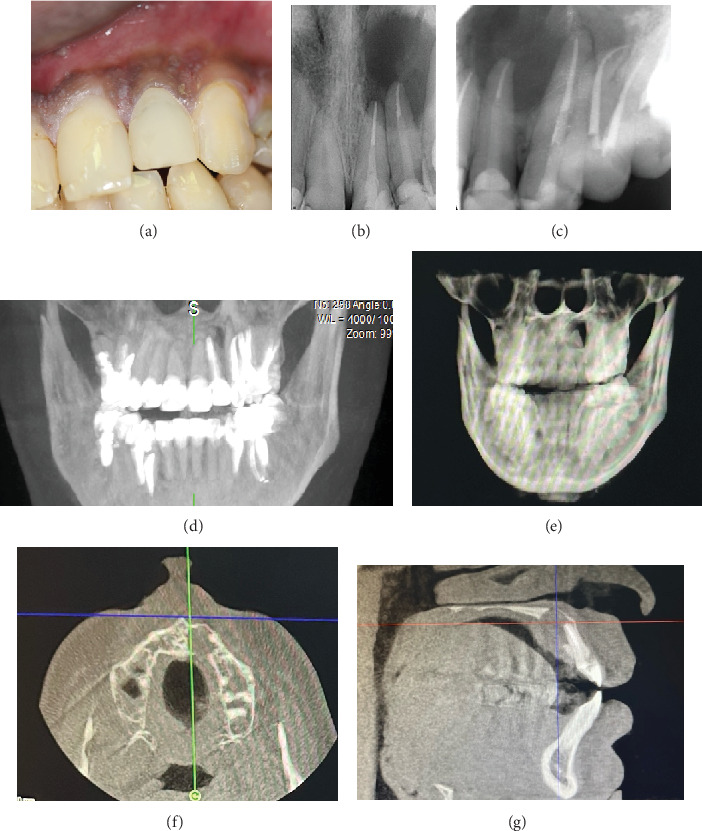
(a) Clinical preoperative photograph. (b, c) Preoperative periapical radiographs (straight and shift views) showing the periradicular lesion involving both Teeth #21 and #22 previously endodontically treated. (d, e) Panoramic image of CBCT of anterior area. (f, g) CBCT slices showing the periradicular through-and-through radiolucency related to treated #21 and #22.

The patient underwent a clinical examination described in detail in Table 1, and radiographic evaluation (panoramic radiograph of the jaws, periapical (PA) radiographs, and a cone beam computed tomography (CBCT) scan), showing a periapical radiolucency over the apices of Teeth #21 and #22 associated with endodontic treatment (Figure 1). Figure 1 details the preoperative status of the case from clinical photographs to radiographic examination (straight and shifted periapical radiographs, CBCT of different planes).

The teeth were tender to percussion and palpation, and both teeth had permanent restorations with good margins and esthetics. The history, clinical, and radiographic examinations determined a diagnosis of a previous nonsurgical RCT with acute symptomatic apical periodontitis.

### 2.1. Treatment Plan

The patient was offered all treatment plan options and decided on EMS. The surgical strategy involves the apicectomy of two roots with retrograde fillings and no bone graft. The procedure was explained to the patient, and signed written consent was obtained. He was also given preoperative nonsteroidal anti-inflammatory drugs (Ibuprofen 400) on the day of the surgery.

### 2.2. Surgical Intervention

The use of a surgical microscope is crucial in modern endodontics, and the American Dental Association (ADA) now mandates that endodontic specialty programs incorporate training on the use of magnification [1]. The microsurgical technique was performed following the guidelines provided by Kim and Kratchman [1] and the use of a Zeiss microscope at high magnification (x16). The mouth was prepared using 0.12% chlorhexidine solution mouthwash for 1 min, and then, the patient was given four capsules of 2% lidocaine with 1:80,000 epinephrine through buccal and palatal infiltration. After the patient was adequately anesthetized, a sharp incision was made into the bone using an 11-blade on a Parker handle. An intrasulcular incision including the dental papilla with two vertical releasing incisions was placed distal to Tooth #25 and distal to Tooth #13, and then, a full-thickness mucoperiosteal flap was elevated buccally, followed by another full-thickness mucoperiosteal flap palatally from Tooth #13 to Tooth #25. The choice of incision technique and flap design was based on the clinical and radiographic parameters [19].

After flap reflection, no bone osteotomy was needed where a breach in both the palatal and buccal cortical plates and root exposure. A round carbide bur with continuous irrigation for cooling was used to refine the bony defect to reach the periapical lesion and the teeth apices. Curettes were utilized to eliminate the soft granulation tissue (size 3 × 5 mm) until healthy bone margins were achieved. The specimen was stored in a formalin solution and sent to the histopathology lab. Hemostasis was accomplished using sterile gauze.

The apical resection was achieved using a high-speed carbide surgical bur (Hu-Friedy, Chicago, Illinois, United States) with a zero-degree bevel for both #21 and #22 [20], and the resected root surface was stained with 1% methylene blue and inspected under high magnification (x16). Ultrasonic surgical JetTips (B&L Biotech USA Inc., Fairfax, Virginia, United States) were used to prepare the root-end, and then the root-end cavity was filled with white mineral trioxide aggregate (MTA) (ProRoot MTA, Dentsply Tulsa Dental, Tulsa, Oklahoma, United States). A radiograph verified the adaptation of the filling material.

**Figure 2:**
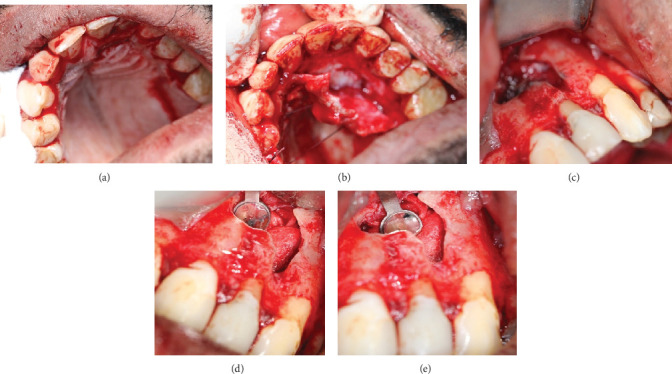
(a–e) Clinical view of the surgical procedure after flap and root exposure.

**Figure 3:**
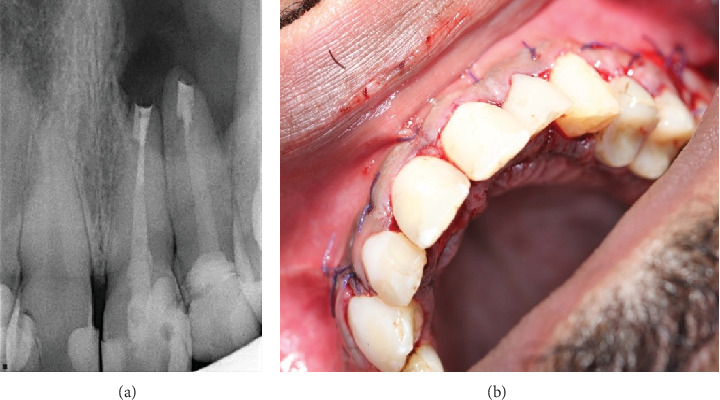
(a, b) Immediate postoperative clinical PA X-ray after placement of retrofill material and postoperative image after flap repositioning and suturing.

Thorough assessment and abundant flushing with normal saline were performed to completely remove root-end filling material and debris that could impede the healing process. A moist gauze was gently applied to reposition the flap [21], and nonresorbable 4-0 vicryl sutures were used in a single interrupted method to secure the flap in place. The patient was given 600 mg of ibuprofen for pain management, along with a broad-spectrum antibiotic (Augmentin) 1 g twice daily for 5 days [22, 23], and the use of 0.2% chlorhexidine mouthwash twice daily for a week (Figures 2 and 3). In Figure 2, the surgical procedure can be visualized, including incisions, buccal and palatal flaps reflections, osteotomy refinement, and 3 mm root resection for both Teeth #21 and #22 followed by retropreparation and retrofill using MTA. Also, Figure 3 entails the immediate postoperative radiographic verifying the retro-fill orientation and placement finalizing with clinical image after flap repositioning and suturing.

**Figure 4:**
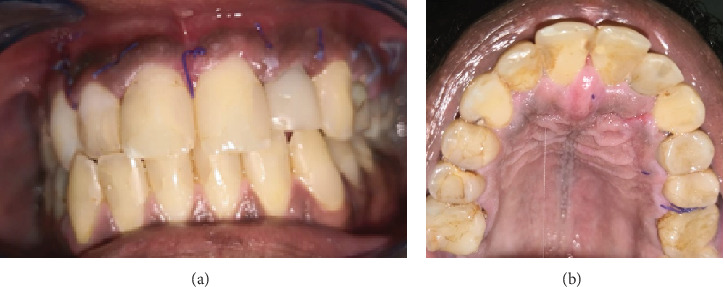
(a, b) 1-week postoperative clinical photograph showing good healing of soft tissue without erosion or necrosis.

**Figure 5:**
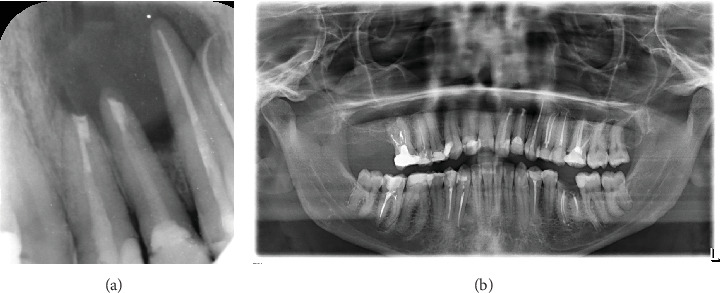
(a, b) 1-year recall radiographic evaluation (periapical and panoramic views).

**Figure 6:**
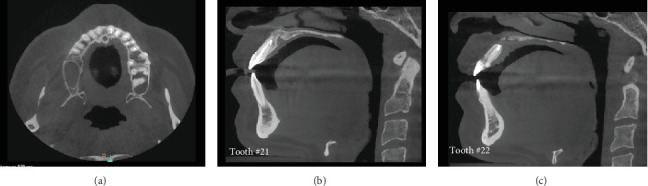
(a–c) 3-year recall CBCT radiographic evaluation of both Teeth #21 and #22; it shows partial healing around the apex of Tooth #21 and incomplete healing around the apex of Tooth #22).

The first follow-up appointment was 1 week postsurgery for suture removal (Figure 4), followed by 1 year (Figure 5) and 3 years (Figure 6) with partial healing around the apex of Tooth #21 and incomplete healing around the apex of Tooth #22. The patient was content, with no clinical signs or symptoms of failure.

## 3. Discussion

The most significant clinical finding in treating this case is that the tooth's integrity and functionality were preserved by the natural healing process of both the alveolar bones and the surrounding tissues. This suggests that, in certain situations, the lack of a bone graft or membrane does not always make the procedure less successful, especially for patients who are in good condition, have undergone appropriate surgery, and are receiving the best postoperative care.

Furthermore, this case indicates that even in a high-stress setting like an Air Force pilot, effective results can be achieved with proper tissue management and surgical site closure without needing further biomaterials.

EMS is the most cost-effective option in most endodontic microsurgical cases, followed by nonsurgical ReRCT with a crown [24]. The preoperative administration of NSAID significantly decreases postoperative pain and swelling [25], and rinsing with 0.12% chlorhexidine gluconate for 1 min decreased the number of microorganisms at the surgical site [25].

A 3-mm root-end amputation was made to eliminate all lateral canals and apical ramifications [1]. To prevent bacterial growth and promote the healing of periapical tissue, the root-end filling material needs to possess specific attributes, such as being biocompatible, maintaining stability in size, being resistant to resorption, and possessing antibacterial properties. Additionally, the material should be easy to manipulate and create a secure seal [26, 27]. Research indicates that MTA often yields superior outcomes compared to other materials [1]. In endodontic MS, MTA is frequently utilized as a retrofill material because of its superior sealing ability, biocompatibility, and radiopacity, all of which reduce microleakage and promote periapical healing [28]. MTA's lengthy setting time, handling challenges, and potential for tooth discoloration are significant disadvantages, nevertheless [29]. According to Camilleri et al. [30], its usage in some therapeutic settings may also be restricted by its comparatively expensive cost when compared to other materials. Because of its biological benefits and long-term performance, MTA is still a dependable option for retrofilling despite these drawbacks.

The rate at which bone lesions heal on radiographic X-rays depends on the defect size, with smaller defects having quicker recovery [31]. The reasons for this are not fully understood, but it is speculated that the interaction between the host and microorganisms could be a contributing factor. It may take longer for the bone to repair, especially if the outer and inner layers have been eroded. It has been reported that traditional endodontic surgery had a low healing rate of 25% for TTLs without apicoectomy or apicoectomy paired with amalgam as root-end-filled material and without the use of a magnification device [32]. However, with the development of EMS, the success rate has improved significantly [10, 33–35] due to advancements in surgical techniques and the enhancement of root-end filling materials [36]. GTR techniques can be used in addition to EMS to enhance bone healing and regeneration, especially in TTLs that affect both cortical plates [37, 38]. However, this is not applicable in our case as the patient was an Air Force pilot who works in a high-altitude environment, which impacts oral health due to the decreased oxygen levels in the blood, causing hypoxia [39, 40]. The high-altitude environment has systemic effects on the human body, leading to negative impacts on bone mass, microstructure, and normal bone biomechanics, ultimately resulting in decreased ability to repair bone defects, with significantly prolonged healing times being a primary issue [41]. Numerous studies have documented oral diseases associated with being at high altitudes and have examined the oral characteristics of living at high altitudes [42, 43] including periodontal inflammation [44, 45], saliva production [46], dental pain and anesthesia [47], inflammatory mediators and bleeding index [48], and dental fluorosis [49]. The effectiveness of bone repair is also notably lowered [50], and the time it takes for a fracture to heal is significantly longer, particularly at high altitudes (5400–6700 m) compared to coastal areas [51]. The impact of hypoxia at high altitudes on periodontal health and inflammation extends beyond the direct impact of hypoxia in the mouth. These effects encompass a decrease in the variety of oral bacteria, a weakened immune response, and changes in the production of inflammatory substances in the mouth [52].

One limitation of not using bone grafting material or tissue engineering in endodontic surgery for TTLs is the potential for slower or less predictable healing. Bone grafts and tissue engineering techniques help promote tissue regeneration and support the healing process by filling voids, stimulating bone growth, and enhancing tissue repair. Without these interventions, healing relies more heavily on the body's natural regenerative capacity, which may be slower or incomplete in some cases. However, in this particular case, healing occurred successfully without the use of grafting material, suggesting that, in certain circumstances, the body can effectively heal through a more conservative approach.

Case success relies on different factors and is determined during follow-up examinations using clinical and radiographic assessments [4, 6, 7]. Success rate evaluation time varied throughout the literature, with some authors claiming that short-term recall has a higher success rate than long-term recall (4-5 years) [53, 54]. However, Pallares-Serrano found that the long-term outcomes (5–9 years) of EMS were less favorable than short-term (1–4 years) outcomes [54]. This inconsistency may be explained by the fact that complex cases require more time for bone healing, particularly with large or TTLs, than cases with minimal or no cortical plate loss. The criteria for evaluating healing were set using Rud and Molven's standards and involved the use of two-dimensional periapical radiography [55, 56]. The case was deemed successful, resulting in fully healed periapical lesions and the absence of any clinical signs or symptoms.

The development of EMS has been made possible by advancements such as the surgical operating microscope, ultrasonic root-end preparation, and calcium silicate materials. Additionally, the use of CBCT has allowed for the development of guided surgical procedures, facilitating precise planning and three-dimensional printing of surgical guides. This has led to increased precision and safety precautions in treatments, with the option for direct flapless access or standard flap access to the osteotomy site [57].

As our understanding of wound healing and tissue engineering improves, so too will our ability to prompt more effective and rapid healing of periapical tissue. By using growth factors and developing new therapies in the future, we might be able to improve periapical healing and produce results that closely resemble the body's natural healing mechanisms. There is a lot of promise for bettering patient care and oral health outcomes with this progression in treatment approaches.

## 4. Conclusion

This case emphasizes how crucial it is to obtain a thorough history and use a multidisciplinary approach to diagnose and manage TTLs, achieving positive outcomes. Using an EMS approach advances the quality of the diagnosis and treatment of teeth with problems of periradicular origin and disease of the apical tissues. Furthermore, infectious diseases (cysts) can be treated without the need for bone graft material, and with good healing of the apical tissues, this approach is also biologically correct for disease management. This emphasizes how crucial it is to combine thorough diagnostic techniques with cutting-edge therapeutic approaches to guarantee efficient management and long-term success in treating complicated dental problems.

## Figures and Tables

**Table 1:** Clinical examination of Teeth #21, #22, and #23.

**Tooth number**	**#21**	**#22**	**#23**
Percussion	++ve	++ve	−ve
Palpation	++ve	++ve	−ve
Probing Depth B side (three readings)	2 2 1	2 2 1	1 1 2
Probing Depth P side (three readings)	1 1 2	1 1 2	2 1 2
Mobility	No	No	No
Biting test	+ve	+ve	−ve
Attached gingival (mm)	2 mm	2 mm	2 mm
Type of coronal restoration	Restoration	Restoration	Restoration
Quality of coronal restoration	Good (consultation done with prostho.)	Good (consultation done with prostho.)	—
Tooth length (mm)	25	25	29
Crown/root ratio	2:3	2:3	2:3

## Data Availability

The data produced and/or examined in this study are not accessible to the public due to ethics approval being granted under the condition that only the researchers involved in the study have access to the identified data. However, the data is available from the corresponding author upon request.
