# Corrigendum to “Healing of Extensive Through-and-Through Periradicular Lesion Using Apicoectomy Without Bone Grafting: A Case Report With 3-Year Recall”

**DOI:** 10.1155/crid/9764587

**Published:** 2025-09-17

Q. Hashem, “Healing of Extensive Through-and-Through Periradicular Lesion Using Apicoectomy Without Bone Grafting: A Case Report With 3-Year Recall”, *Case Reports in Dentistry* 2025 (2025), 7225338, https://doi.org/10.1155/crid/7225338.

In the article titled “Healing of Extensive Through-and-Through Periradicular Lesion Using Apicoectomy Without Bone Grafting: A Case Report With 3-Year Recall,” author Qamar Hashem was affiliated to “Conservative Dentistry Department, College of Dentistry, Prince Sattam Bin Abdulaziz University, Al-Kharj, Saudi Arabia” which is incorrect. The correct affiliation for this author is as follows:

*Endodontic Division, Department of Conservative Dental Sciences, College of Dentistry, Princes Sattam Bin Abdulaziz University, Al-Kharj, 11942, Saudi Arabia*

We apologize for this error.

